# Effect of policosanol from insect wax on amyloid β-peptide-induced toxicity in a transgenic *Caenorhabditis elegans* model of Alzheimer’s disease

**DOI:** 10.1186/s12906-021-03278-2

**Published:** 2021-03-30

**Authors:** Xin Zhang, Chenjing Ma, Long Sun, Zhao He, Ying Feng, Xian Li, Jin Gan, Xiaoming Chen

**Affiliations:** The Key Laboratory of Cultivating and Utilization of Resource Insects of State Forestry Administration, Research Institute of Resource Insects, Chinese Academy of Forestry, Kunming, 650224 China

**Keywords:** Alzheimer’s disease, Insect wax, Policosanol, β-Amyloid, *C. elegans*, CL4176

## Abstract

**Background:**

Alzheimer’s disease (AD), an age-related neurodegenerative disorder and a serious public health concern, is mainly caused by β-amyloid (Aβ)-induced toxicity. Currently, a limited number of drugs are effective against AD, and only a few are used for its treatment. According to traditional Chinese medicine, white wax is mainly composed of policosanol, hexacosanol, and octacosanol. Policosanol has been shown to reduce lipid levels in blood and alleviate the symptoms associated with diabetic complications and neurodegenerative disorders, such as Parkinson’s disease and AD. However, the efficacy of policosanol depends on the purity and composition of the preparation, and the therapeutic efficacy of policosanol derived from insect wax (PIW) in AD is unknown.

**Methods:**

Here, we identified the main components of PIW and investigated the effects of PIW on Aβ-induced toxicity and life-span in a transgenic *Caenorhabditis elegans* model of AD, CL4176. Furthermore, we estimated the expression of amyloid precursor-like protein (*apl-1*) and the genes involved in various pathways associated with longevity and alleviation of AD-related symptoms in PIW-fed CL4176.

**Results:**

PIW mainly consists of tetracosanol, hexacosanol, octacosanol, and triacontanol; it could decrease the Aβ-induced paralysis rate from 86.87 to 66.97% (*P* < 0.01) and extend the life-span from 6.2 d to 7.8 d (*P* < 0.001) in CL4176 worms. Furthermore, PIW downregulated *apl-1*, a gene known to be associated with the levels of Aβ deposits in *C. elegans*. Additionally, our results showed that PIW modulated the expression of genes associated with longevity-related pathways such as heat shock response, anti-oxidative stress, and glutamine cysteine synthetase.

**Conclusion:**

Our findings suggest that PIW may be a potential therapeutic agent for the prevention and treatment of AD. However, its effects on murine models and patients with AD need to be explored further.

**Supplementary Information:**

The online version contains supplementary material available at 10.1186/s12906-021-03278-2.

## Background

**Fig. 1:**
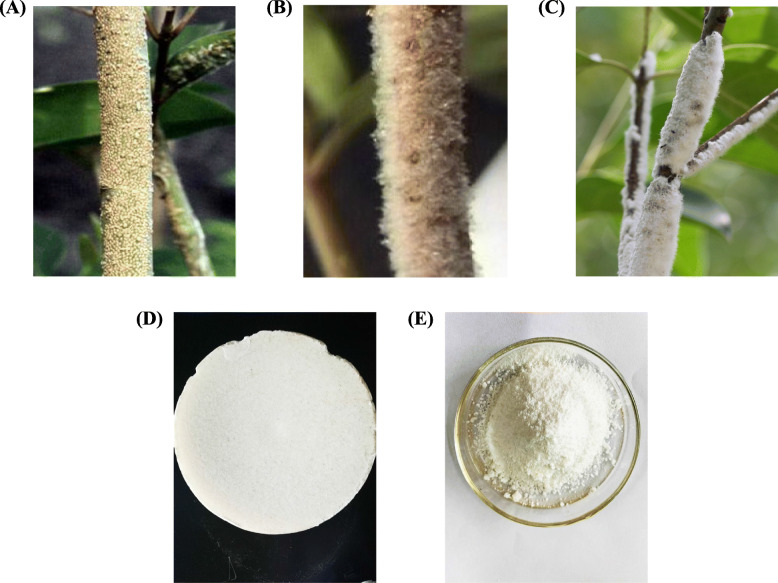
Morphology of insect wax and the extracted policosanol. **a** The second-instar male nymphs of *Ericerus pela* Chavannes. **b** The second-instar male nymphs of *Ericerus pela* begin to secrete wax. **c** Secreted wax layer*.* **d** White wax. **e** Powdery form of policosanol extracted from insect wax using the reduction method

Insect wax secreted by the male second-instar larva of the scale insect *Ericerus pela* Chavannes (Fig. 1a, Fig. 1b, Fig. 1c), also known as white wax (Fig. 1d) [1], has been used in China for over a thousand years for various medicinal and industrial purposes [2, 3]. It is extensively used in the textile and batik, food packaging, precision instrument lubrication and sealing, medicine, and cosmetic industries [1, 4, 5]. *E. pela* is found in the north subtropical, middle subtropical, and temperate regions, including China, Vietnam, Japan, and North Korea. In China, it is mainly found in the Yunnan, Guizhou, Sichuan, Zhejiang, Shaanxi, Guangxi, and Hunan provinces. Owing to the various applications of white wax, breeding of the scale insect is vital to the white wax bio-industry in these regions. Chinese white wax has been a featured bio-industry product, and hundreds of tons of the insect wax is produced every year in China [1]. The primary component of insect wax is high molecular weight wax esters, consisting of monobasic saturated fatty alcohol and monobasic saturated fatty acids, accounting for about 93–95% of its constituents [6]. Hexacosanoic acid hexacosyl ester and octocosoic acid dioctadecyl ester are the major wax esters in insect wax [7]. As described in Li Shizhen’s “Ben Cao Gan Mu” (Compendium of Materia Medica), insect wax is nontoxic and can be used as a hemostatic agent for bones and muscles, to relieve pain and reinforce deficiencies, to promote concrescence of fractures, and to treat alopecia of the scalp [8]. As demonstrated by recent studies, Chinese white wax scale modulates humoral and cellular immunity, has antioxidant activities, improves immune response in vivo, and attenuates atopic dermatitis [9–13]. In China, research on white wax and the white wax scale insect has been continually carried out to promote the growth of its insect wax industry.

Policosanol derived from insect wax (PIW) is a mixture of saturated monohydric alcohols. It is a white powdery substance (Fig. 1e) obtained by reducing white wax at yields of 95–98% [14]. Policosanols mainly exist as fatty acid esters in the natural sources, such as bees wax, sugar cane wax, rice bran wax, white wax, corn bran wax, wheat germ, epidermis of some plants, rhizome, and grains [15–18]. Researchers from Cuba have isolated and produced the first policosanol supplement from sugarcane wax [19]. The beneficial physiological effects of policosanols include reducing platelet aggregation, endothelial damage, and foam cell formation [20], increasing muscle endurance [21], improving performance of coronary heart disease patients during exercise [22], and anti-arthritic and antioxidant properties [23]. Furthermore, studies exploring the physiological activities of policosanol have focused on its role in decreasing the levels of low-density lipoprotein (LDL) and increasing the levels of high-density lipoprotein (HDL) in the blood [24–31]. In addition, PIW promotes skin wound healing in mice; exhibits antibacterial, anti-inflammatory, and analgesic properties; and is skin-safe [32]. Furthermore, it has been reported to cause proliferation of human follicle dermal papilla cells [33] and promote hair growth in androgen-induced alopecia mice [34]. However, the efficacy of policosanols depends on the purity and composition of the preparation [19]; hence, policosanols obtained from different sources may have varied effects. PIW is composed of hexacosanol, octacosanol, tetradecanol, triacontanol, pentadecanol, and heptanol [14]. Hexacosanol and octacosanol have been reported to reduce the levels of lipids in the blood and alleviate the symptoms associated with diabetic complications and neurodegenerative disorders, such as Parkinson’s and Alzheimer’s disease (AD) [35–37].

AD is a progressive neurodegenerative disorder and is the most common cause of dementia in the aged population [38]. The major constituent of senile plaques, the 39–43 amino acid-long amyloid-beta peptide (Aβ), is thought to be central to the pathogenesis of AD [39, 40]. Currently, the number of drugs effective against AD is limited and only a few drugs, including acetylcholinesterase inhibitors and N-methyl-D-aspartate receptor antagonists, have been approved by the U.S. Food and Drug Administration for the treatment of AD [41, 42]. However, these drugs provide short-term relief to AD patients with mild to moderate symptoms and have obvious side effects [43]. Hence, efforts have been made to develop strategies that target Aβ for the prevention and treatment of AD [44, 45]. Nonetheless, it is particularly important to explore natural sources for drugs with fewer side effects and high efficacy.

In the current study, we investigated the effects of PIW on Aβ-induced toxicity using a transgenic *Caenorhabditis elegans* strain, CL4176. Additionally, we explored various pathways associated with the alleviation of paralysis, along with their underlying mechanisms, as well as discussed the potential of PIW as a treatment option for AD.

## Methods

### Preparation of policosanol from white wax and quantification of PIW using gas chromatography (GC)

White wax was purchased from a market in the Zhijiang county, Hunan province in the east of China. Policosanol was prepared by reduction of white wax using lithium aluminum hydride (LiAlH_4_), as described previously [14]. The ratio of wax and LiAlH4 was 100:5 ~ 7(w: w) and the reaction was carried out in a 250 mL flask. Briefly, white wax and LiAlH_4_ were allowed to react in a flask, after which tetrahydrofuran was added slowly at 60 °C with ultrasonication (40 KHz). This was followed by neutralization of tetrahydrofuran with 1 mol/L of chlorohydric acid and washes with hot water; the resultant product was then dried at 65 °C. Finally, the solid residue was recrystallized using a solution of chloroform and absolute ethanol (v/v, 3:1), followed by drying at 65 °C. The w:w ratio of the solid residue to the mixture of chloroform and absolute ethanol was 1:10, and the obtained yield of PIW was 95–98%.

Four standard components— 20.9 mg of tetracosanol, 20.1 mg of hexacosanol, 20.9 mg of octacosanol, and 20.6 mg of triacontanol (Sigma-Aldrich)—were dissolved in 100 mL chloroform, using ultrasonication, to prepare a standard solution. Then, 0.50, 1.00, 2.50, 5.00, and 10.00 mL of the standard solution was added into five 10 mL volumetric flasks, followed by the addition of chloroform, to prepare a gradient of standard working solutions for further use. PIW (16.0 mg) was dissolved in chloroform to prepare a sample solution with a concentration of 1.6 mg/ml. After filtration through a 0.45 μm organic film (PVDF, Millipore), 2 μL of each working solution and the sample solution was injected into the Agilent Gas Chromatograph instrument (7809B). The column used was HP-5 (30 m × 0.25 mm × 0.25 μm), with the following parameters: injector temperature; 320 °C, detector temperature: 340 °C, column oven temperature: 200 °C (1 min), increased to 320 °C (10 min) at a rate of 5 °C/min, carrier gas: nitrogen, purity ≥99.99%, fuel gas: hydrogen, purity ≥99.99%, column flow rate: 1.0 mL/min, hydrogen flow rate: 30 mL/min, air flow rate: 300 mL/min. The contents of PIW were calculated using the peak area of the four standard components and sample solution.

### *C. elegans* strains and maintenance conditions

CL4176, the transgenic *C. elegans* strain, was provided by Dr. Ding Aijun, Kunming University, Yunnan, China. The strain was maintained at 16 °C on nematode growth media (NGM) plates seeded with *E. coli* OP50, and the temperature was increased to the permissive 25 °C to activate the expression of human Aβ_1–42_ peptide and induce paralysis. *E. coli* OP50 was grown in Luria–Bertani growth medium. The synchronization of nematode culture was achieved by sodium hypochlorite treatment, which kills adult worms, and the hatched L1 larvae were recovered on a NGM plate. The worms, either from the L1 stage (1 d of age) or egg stage, were fed the drugs.

### Paralysis assay

The synchronized nematode hatchlings derived from the synchronized eggs (day 1) were cultured in M9 buffer at 16 °C. Worms at stage L1 were fed 0.5, 1, 2, 3, or 4 μg/mL of PIW, whereas unfed worms were considered as controls. The concentrations of PIW were decided based on the results of a preliminary study(data not shown). Meanwhile, the temperature was increased to 25 °C at start of the 36th hour, which represents the 3rd larval stage, and was maintained at 25 °C until the end of the paralysis assay. Paralysis rates of the control group and different treatments were scored at the 48th hour, which represents the late period of the 4th larval stage. Worms that did not move their whole body or only moved their head when gently touched with a platinum loop, were scored as paralyzed. Each group had at least 100 worms.

### Life-span analysis

Worms were fed PIW (2 or 3 μg/mL) from stage L1, and late 4th stage larvae were synchronized. Untreated worms were considered as controls. Day 1 of the life-span assay started at 24 h after transferring the worms onto new plates. The number of worms was counted every day until the last worm died. Worms that did not respond to 3 to 5 mechanical touches were scored as dead and were removed from the plates. Furthermore, the worms that died from crawling off the agar were excluded from the analysis.

### Measurement of superoxide dismutase (SOD) and reactive oxygen species (ROS) levels

Intracellular SOD levels were measured in transgenic CL4176 worms using a SOD kit (Nanjing Jiancheng Bioengineering Institute, Jiangsu, China). Age-synchronized, L1 stage *C. elegans* were transferred on to NGM plates containing 2 or 3 μg/mL of PIW and incubated for 48 h at 25 °C. The worms were then collected into a microfuge tube and washed twice with phosphate-buffered saline (PBS) to remove *E. coli.* Then, 200 μL of PBS was added to the homogenate on ice, centrifuged, and the resultant supernatant was used to measure SOD activity.

Intracellular ROS levels were measured using the 2′,7′-dichlorodihydro-fluorescein diacetate (H2DCF-DA) fluorescent probe (Nanjing Jiancheng Bioengineering Institute, Jiangsu, China). After 48 h of incubation, the worms were collected into a microfuge tube and washed thrice with M9 buffer, ground, and transferred to 96-well plates. Then, 50 μL of H2DCF-DA was added to each well and a final concentration of 50 μmol/L of H2DCF-DA was obtained. The fluorescence intensity of (dichloro) fluorescein was measured at 640 nm using the fluorescence enzyme method, and the concentration of ROS was determined.

### Quantitative reverse transcription (qRT)-PCR analysis

Worms were synchronized on NGM plates with 2 or 3 μg/mL of PIW after culturing for 2 d. Untreated nematodes were used as controls. Total RNA was isolated using the TRIzol reagent (Invitrogen, USA) as per manufacturer’s protocol. Equal amounts of RNA were reverse transcribed into cDNA with the PrimeScript™ RT reagent kit (Takara, Japan). Primers used for amplification, along with their corresponding gene names, are listed in Table 1. qRT PCR was performed on the Applied Biosystems QuantStudio 3 (ABI, USA) using 20 μL of reaction mixture containing primers, cDNA, and PowerUp™ SYBR Green Master Mix (ABI, USA). The PCR amplification of 40 cycles was carried out at two stages; the hold stage involving UDG activation at 50 °C for 2 min and Dual-Lock TM DNA at 95 °C for 2 min, and the PCR stage involving denaturation at 95 °C for 15 s and annealing and extension at 60 °C for 1 min. Relative quantification of the genes was performed using the 2^-ΔΔCT^ method, and *act-1* was used as the internal standard.

**Table 1:** Primer sequences used for amplification of genes in policosanol derived from insect wax (PIW)-fed transgenic *Caenorhabditis elegans*

Gene name	Primer type	Primer sequence
act-1	Forward	5′-ACTGAAGCCCCACTCAATCC-3’
	Reverse	5′-GACATACATGGCTGGGGTGT-3’
apl-1	Forward	5′-AGGTGATGAAGGAGTGGGGA-3’
	Reverse	5′-AACTTCTCGGCTCCCTTTGG-3’
bcat-1	Forward	5′-GGATTCCAGCCAGTCAGCTT-3’
	Reverse	5′-CCAGATTGTTGGGGCGTAGT-3’
daf-16	Forward	5′-CATCATCTTTCCGTCCCCGA-3’
	Reverse	5′-AGCTGGAGAAACACGAGACG-3’
sod-3	Forward	5′-CCAACCAGCGCTGAAATTCAATGG-3’
	Reverse	5′-GGAACCGAAGTCGCGCTTAATAGT-3’
lipl-4	Forward	5′-TGCTCACGGCGTGTTCTTAT-3’
	Reverse	5′-AGTTCATCGGACCCATGTTTCT-3’
lips-17	Forward	5′-GATTCAAGTGGTTGCTGCGT-3’
	Reverse	5′-AAGCTCCTCCGAGAATTGCC-3’
hsf-1	Forward	5′-TTGACGACGACAAGCTTCCAGT-3’
	Reverse	5′-TTGACGACGACAAGCTTCCAGT-3’
hsp-16.2	Forward	5′-GGTGCAGTTGCTTCGAATCTT-3’
	Reverse	5′-TTCTCTTCGACGATTGCCTGT-3’
skn-1	Forward	5′-GTGGATCACGCTACCAACGA-3’
	Reverse	5′-CTGGCCAGTGGAACAACTCT-3’
gcs-1	Forward	5′-ATTCGGAATGGGGTGCTGTT-3’
	Reverse	5′- TCGGTGTAATCGGTGTCAGC-3’

### Statistical analysis

Statistical analysis was performed using the GraphPad Prism 8.0 software. All values were presented as means ± standard deviation. One-way analysis of variance was used for determining the statistically significant differences between groups, and a *P* value < 0.05 was considered significant. Three independent experiments were conducted, and all the experiments were performed in triplicates.

## Results

### Analysis of the main components of PIW

**Fig. 2:**
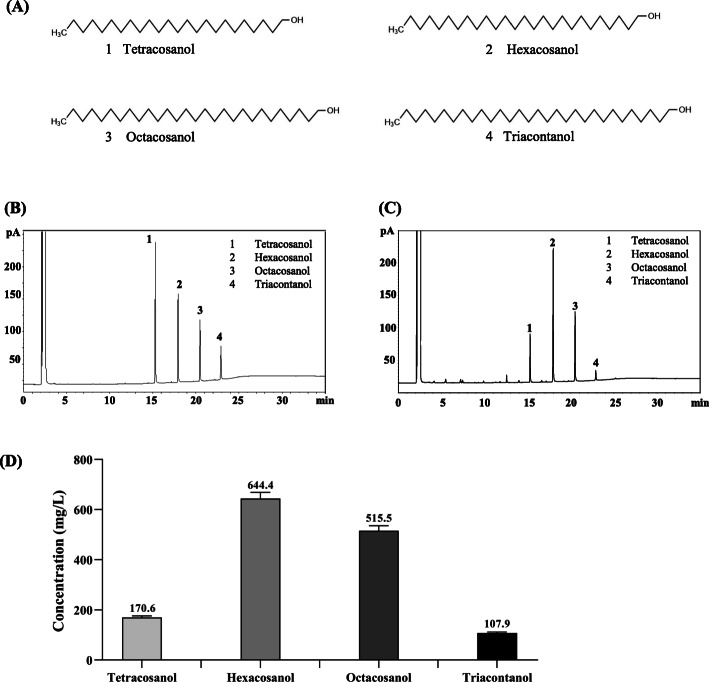
Composition analysis of policosanol derived from insect wax (PIW). **a** Chemical structures of tetracosanol, hexacosanol, octacosanol and triacontanol. **b** Chromatogram of standard compounds (200 mg/L) obtained using gas chromatography (GC). **c** Chromatogram of PIW obtained using GC. **d** Concentrations of the different components of PIW

GC analysis showed that PIW mainly consists of four compounds, namely tetracosanol, hexacosanol, octacosanol, and triacontanol (Fig. 2a, Fig. 2b). Their concentration in the obtained PIW was 170.6 mg/L of tetracosanol, 644.4 mg/L of hexacosanol, 515.5 mg/L of octacosanol, and 170.9 mg/L of triacontanol (Fig. 2c, Fig. 2d).

### Paralysis assay of PIW-fed transgenic *C. elegans*

**Fig. 3:**
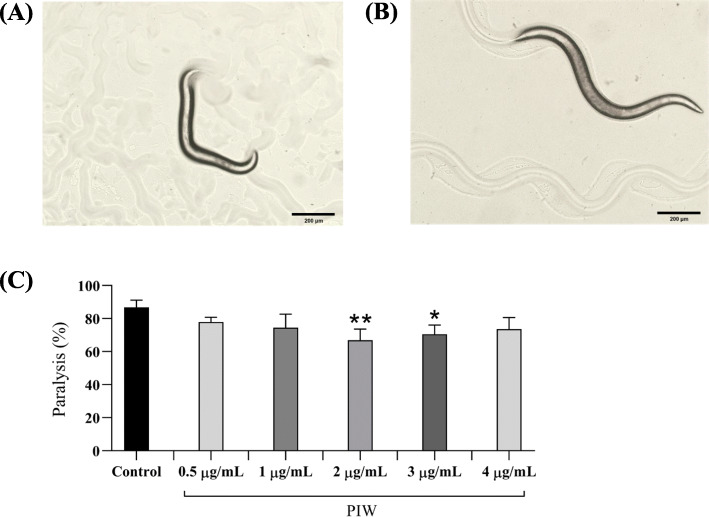
Paralysis assay of the *Caenorhabditis elegans* strain CL4176 fed varying concentrations of policosanol derived from insect wax (PIW). **a** Representative image of a paralyzed CL4176 worm. **b** Representative image of a non-paralyzed CL4176 worm. **c** Paralysis rate of the CL4176 worms fed different concentrations of PIW, and control worms at 48 h after temperature elevation. *P < 0.05 and **P < 0.01 compared to those in the untreated control

We investigated the protective role played by PIW against Aβ-induced toxicity using the transgenic *C. elegans* strain, CL4176. Our results indicated that the CL4176 worms fed PIW showed a reduced rate of paralysis compared to that of control worms. PIW treatment also rescued paralysis in worms (Fig. 3a) and allowed complete sinusoidal motion (Fig. 3b). However, this restoration by PIW was not dose-dependent. Furthermore, PIW at 2 μg/mL concentration was found to be the most effective dose, wherein the rate of paralysis was reduced from 86.87% (control worms) to 66.97% at 48 h after temperature elevation (Fig. 3c). Based on these results, we used concentrations of 2 and 3 μg/mL of PIW in the subsequent experiments.

### Life-span analysis of PIW-fed transgenic *C. elegans*

**Fig. 4:**
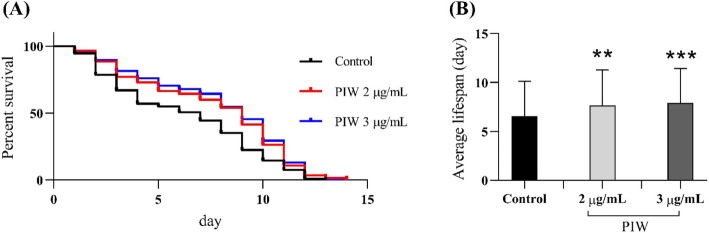
Life-span assay of the transgenic *Caenorhabditis elegans* strain CL4176 fed 2 or 3 μg/mL of policosanol derived from insect wax (PIW). **a** Survival curves of worms from the two treatment groups and the control group. **b** Average survival rate of worms from the two treatment groups and the control group. **P < 0.01 and ***P < 0.001 compared to those in the untreated control group

Life-span analysis showed that PIW treatment significantly increased the survival rate of the transgenic worms (Fig. 4a). Furthermore, a significant (*P* value < 0.001) increase in the mean life-span of PIW-fed worms, compared to that of the control group, was observed (Fig. 4b). The average life-span of the control group was 6.2 d, whereas that of the PIW-fed (3 μg/mL) group was 7.8 d.

### SOD and ROS levels in PIW-fed transgenic *C. elegans* strain CL4176

**Fig. 5:**
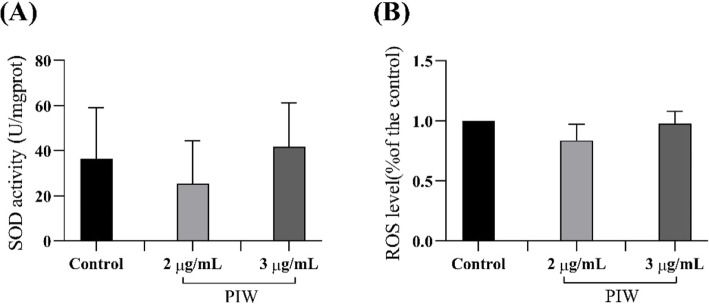
Levels of superoxide dismutase (SOD) and reactive oxygen species (ROS) in policosanol derived from insect wax (PIW)-fed (2 or 3 μg/mL) *Caenorhabditis elegans* strain CL4176. **a** SOD and in PIW-fed (2 or 3 μg/mL) the *C. elegans* strain CL4176, 48 h after temperature elevation. **b** ROS in PIW-fed (2 or 3 μg/mL) the *C. elegans* strain CL4176, 48 h after temperature elevation

Our results demonstrated that PIW treatment decreased and increased the SOD levels at 2 and 3 μg/mL concentrations, respectively, compared to that in the control group; however, these changes were statistically insignificant (Fig. 5a). Similarly, PIW treatment in the *C. elegans* strain CL4176 had no effect on ROS levels (Fig. 5b).

### Relative mRNA expression levels in PIW-fed transgenic *C. elegans* strain CL4176

**Fig. 6:**
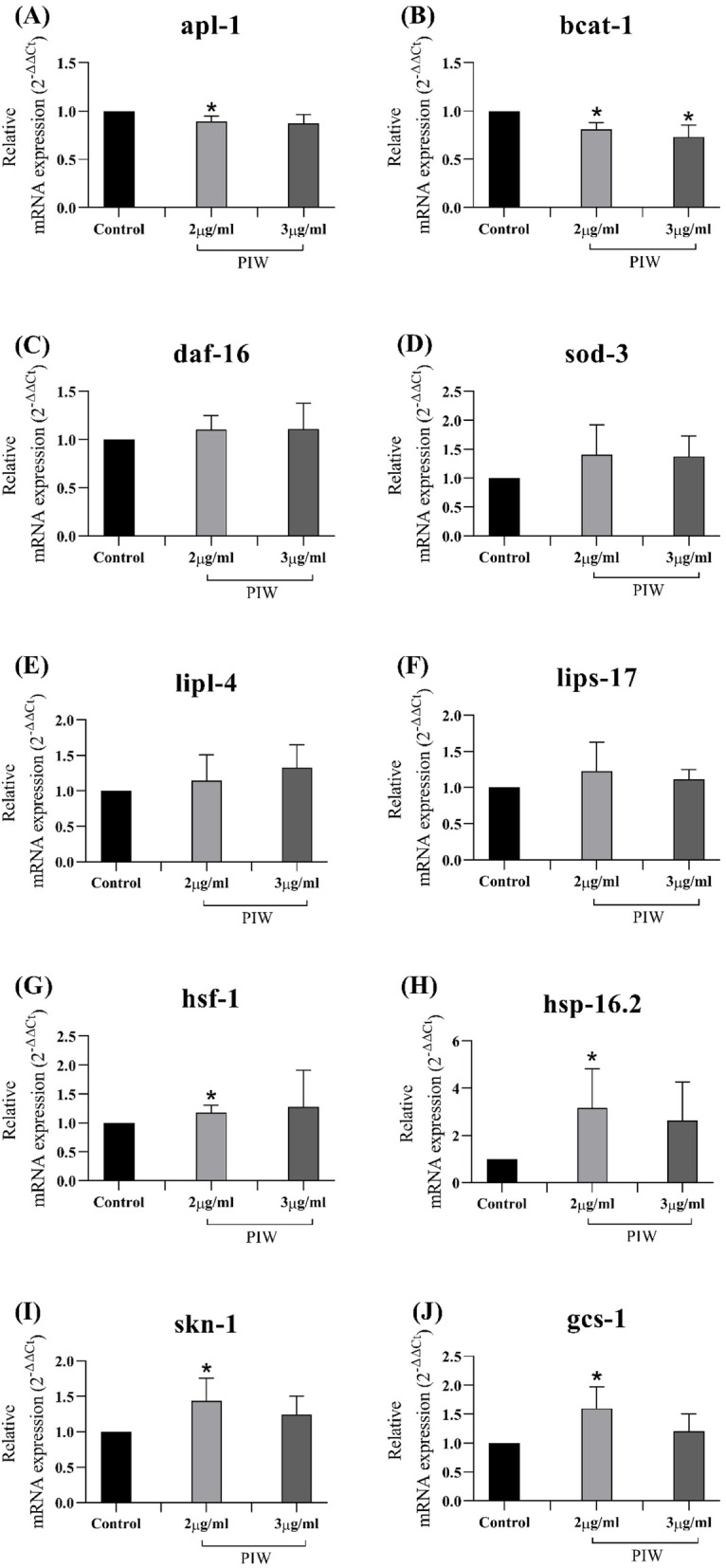
Effect of policosanol derived from insect wax (PIW) on the relative expression of genes in CL4176. Relative expression of the genes involved in various pathways associated with longevity in the transgenic *Caenorhabditis elegans* strain CL4176*.* Expression levels of (**a**) *apl-1* (**b**) *bcat-1* (**c**) *daf-16* (**d**) *sod-3* (**e**) *lipl-4* (**f**) *lips-17* (**g**) *hsf-1* (**h**) *hsp-16.2* (**i**) *skn-1*, and (**j**) *gcs-1*. *P < 0.05 compared to that in the control group

We estimated the expression levels of amyloid precursor-like protein (*apl-1*) and the genes involved in various pathways associated with longevity and alleviation of AD-related symptoms, in the PIW-fed transgenic *C. elegans* strain CL4176. The expression of genes involved in redox homeostasis (*daf-16* and *sod-3*), heat shock response (*hsf-1* and *hsp-16.2*), anti-oxidative stress (*skn-1* and *gcs-1*), lipid metabolism (*lips-17* and *lipl-4*), and branched-chain amino acids (BCAAs) metabolism (*bcat-1*) was estimated. The results showed that PIW downregulated the expression of *apl-1* (0.87 ± 0.05 fold; *P* < 0.05) (Fig. 6a), which is the only ortholog of human amyloid precursor protein (APP), and *bcat-1*(0.73 ± 0.12 fold; *P* < 0.05) (Fig. 6b). However, PIW treatment had no significant effect on the expression of *daf-16, sod-3*, *lipl-4*, and *lips-17* (Fig. 6c-f), whereas, it significantly upregulated *hsf-1*, *hsp-16.2*, *skn-1*, and *gcs-1* (1.18 ± 0.12 fold; 3.18 ± 1.64 fold; 1.44 ± 0.31 fold; 1.60 ± 0.37 fold; *P* < 0.05) (Fig. 6g-j).

## Discussion

**Fig. 7:**
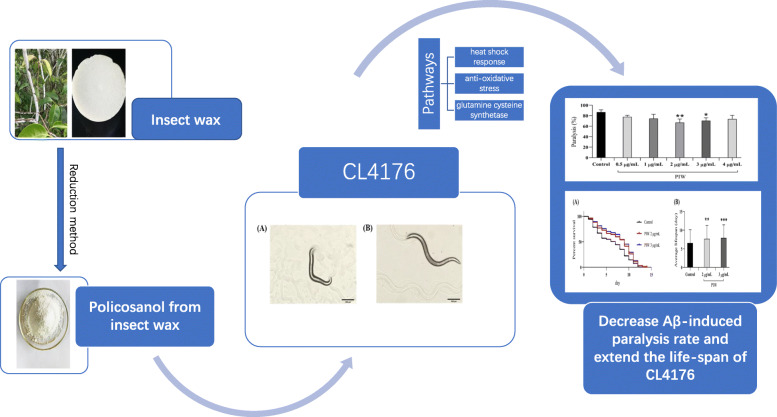
Flow-chart summary of the article

In this study, we employed a transgenic *C. elegans* AD model (CL4176) to evaluate the pharmacological effects of PIW on counteracting Aβ-induced toxicity (Fig. 7). Our results indicate that PIW alleviates paralysis by decreasing the levels of amyloid formation and extending the lifespan of the transgenic *C. elegans* by modulating the pathways associated with heat shock response, anti-oxidative stress, and glutamine cysteine synthetase.

CL4176 is a commonly used in vivo model for the experimental evaluation of drugs for the treatment of AD [46, 47]. It has been used to study the effects of *Ginkgo biloba* extract EGb761 [48], traditional Chinese medicine Liu Wei Di Huang Wan [49], tetracycline [50], and lignans from the roots of *Acorus tatarinowii* [51], against Aβ-induced toxicity. The studies exploring the longevity and alleviation of paralysis in CL4176 have mainly focused on pathways related to redox homeostasis, heat shock response, oxidative stress, and insulin signaling [49, 52–56]. Previous studies demonstrated that the active substances mainly function by targeting the redox homeostasis and anti-oxidative stress pathways in the transgenic *C. elegans* AD model, and alleviate paralysis by increasing the activity of antioxidant enzymes, such as SOD and catalase, and upregulating the expression of *daf-16*, *ctl-1*, *hsp-16.2*, *sod-3*, and *sir-2.1* [49, 54, 55, 57–59]. Our study showed that PIW treatment led to the downregulation of *apl-1*, indicating a reduction in the AD-associated aggregation of APP [60–62]. However, PIW treatment had no significant effect on SOD and the expression of related genes in transgenic *C. elegans*; hence, the reduction in Aβ protein aggregation may be attributed to the induced expression of the genes related to heat shock response, anti-oxidative stress, and glutamine cysteine synthetase pathways. The heat shock factor HSF-1 is known to regulate protein folding and gene expression in response to heat stress and is associated with longevity [63]. Furthermore, it acts as a transcription factor for the genes associated with longevity and Aβ-induced toxicity in transgenic *C. elegans* [52]. Small heat shock proteins are low molecular weight polypeptides with chaperone-like activity that increase the survival of *C. elegans* under stress, and are induced by Aβ expression [64]. Since the heat shock response of *C. elegans* is controlled by neurons, hsp-16.2, which is induced by Aβ protein, protects against the abnormal accumulation of toxic proteins [65]. Furthermore, the overexpression of hsp-16.2 is known to inhibit Aβ-induced toxicity [66]. In our study, PIW enhanced the mRNA levels of *hsf-1* and its classical target gene *hsp-16.2*, indicating that PIW can regulate the transcriptional activity of hsf-1, which may explain the counteracting effect of PIW against Aβ-induced cytotoxicity. Oxidative stress is one of the key factors in the process of ageing and has been reported to play a crucial role in the pathophysiology of AD [67, 68]. Additionally, it has been shown that oxidative stress-induced damages often occur near amyloid plaques in the brain tissues of AD patients [69]. The upregulation of *skn-1* and *gcs-1* in our study suggested that the elevation of antioxidant stress in transgenic *C. elegans* may be one of the mechanisms that protect it from Aβ-induced toxicity. In the brain, the glutamate level is tightly regulated through metabolite exchange in neuronal, astrocytic, and endothelial cells. In the brain of an AD patient, Aβ can interrupt the effective uptake of glutamate by astrocytes, in turn evoking a cascade of events that leads to neuronal swelling, destruction of membrane integrity, and ultimately cell death [70]. The branched chain aminotransferase (BCAT) enzyme plays an integral role in regulating the brain glutamate levels [71]. In AD patients, an increase in the level of BCAT has been reported in the hippocampus [72]. Furthermore, the altered expression of *bcat-1* in *C. elegans* leads to increase in the levels of BCAAs, which has been reported to promote longevity in *C. elegans* [73]. Therefore, downregulation of *bcat-1* by PIW treatment in our study may have played a role in the remission of AD-related symptoms. Hence, a detailed study on the BCAA metabolism pathway may provide a novel approach for the treatment of AD. For decades, treatment strategies related to AD have focused on the amelioration of Aβ toxicity and promotion of longevity-related pathways. However, these treatment strategies are challenging and have failed to demonstrate efficacy. Although a few studies have indicated the importance of glutamate pathway in the brain of an AD patient [70], it has been not been explored as a treatment option for AD. We found that PIW can reduce the expression of *bcat-1*, which has been reported to be linked to various pathological states, including cell proliferation [74], decreased survival of septic mice [75], and increased accumulation of liver fat [76]. Although our study demonstrates the effect of PIW on the glutamate metabolism pathway in the transgenic *C. elegans* strain CL4176 and may help in drug development, further studies are required to identify the detailed underlying mechanisms.

## Conclusion

In conclusion, the efficacy of policosanol depends on the source, purity, and composition of the preparation. We explored the effect of PIW treatment on alleviation of AD-related symptoms and associated preliminary mechanisms. Additionally, for the first time, we investigated the role of PIW in reducing Aβ-induced toxicity in the transgenic *C. elegans* strain CL4176. We showed that policosanol, which is generally thought to reduce LDL and increase HDL cholesterol levels in the blood, had no significant effect on the expression of lipid metabolism-related genes (*lips-17* and *lipl-4*) in the transgenic AD model. Furthermore, PIW reduced the Aβ-induced toxicity in transgenic *C. elegans* by upregulating the expression of genes related to heat shock response and oxidative stress, and downregulating the expression of *bcat-1*, which is involved in the glutamine cysteine synthetase pathway and should be explored further as a drug target in AD. In the future, we will conduct the pharmacodynamic study of each component of PIW, including tetracosanol, hexacosanol, octacosanol, and triacontanol. Nevertheless, the effectiveness of PIW needs to be explored in murine models and humans.

## Supplementary Information

**Additional file 1.** Results of preliminary experiments

## Data Availability

The data is available and will be provided upon request to the corresponding author.

## Abbreviations

AD: Alzheimer’s disease
PIW: Policosanol derived from insect wax
Aβ: β-amyloid
PVDF: Poly (1,1-difluoroethylene)
LDL: Low-density lipoprotein
HDL: High-density lipoprotein
NGM: Nematode growth medium
SOD: Superoxide dismutase
ROS: Reactive oxygen species
PBS: Phosphate-buffered saline
H2DCF-DA: 2′,7′-dichlorodihydro-fluorescein diacetate
BCAT: Branched chain aminotransferase
BCAAs: Branched-chain amino acids
