# Supplementary material for: Effect of policosanol from insect wax on amyloid β-peptide-induced toxicity in a transgenic Caenorhabditis elegans model of Alzheimer’s disease
Source: BMC Complement Med Ther. 2021 Mar 30;21:103. doi: 10.1186/s12906-021-03278-2 (PMC8011155; doi:10.1186/s12906-021-03278-2)

Results of preliminary experiments

**F**i**gure 1**

Paralysis rate of the transgenic *Caenorhabditis elegans* strain CL4176 worms fed different concentrations of policosanol derived from insect wax (PIW), solvent control, and control worms at 48 h after temperature elevation. **P* < 0.05 and ***P* < 0.01 compared to those in the untreated control.


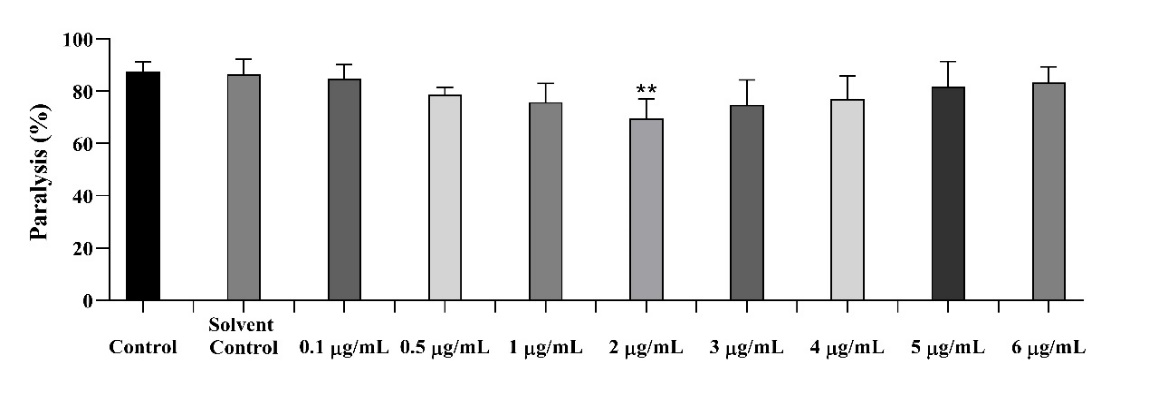


**F**i**gure** **2**

Survival curves of the transgenic *Caenorhabditis elegans* strain CL4176 fed with different concentrations of policosanol derived from insect wax (PIW).


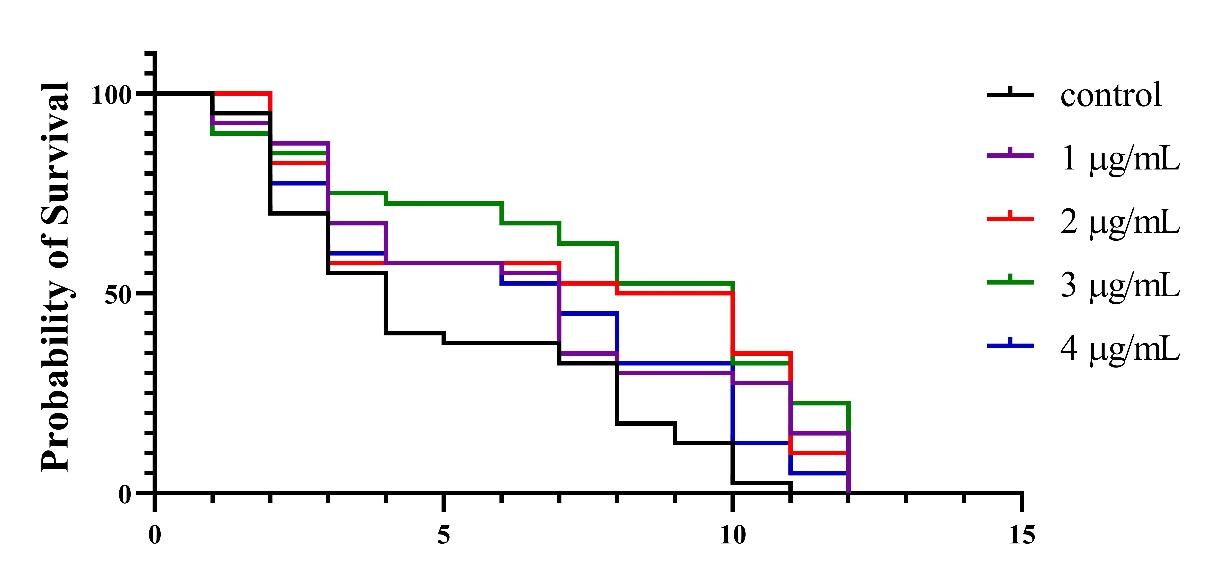

Supplement: Supplementary file 1 — Additional file 1. Results of preliminary experiments [file 12906_2021_3278_MOESM1_ESM.docx]
